# An MR Brain Images Classifier System via Particle Swarm Optimization and Kernel Support Vector Machine

**DOI:** 10.1155/2013/130134

**Published:** 2013-09-16

**Authors:** Yudong Zhang, Shuihua Wang, Genlin Ji, Zhengchao Dong

**Affiliations:** ^1^School of Computer Science and Technology, Nanjing Normal University, Nanjing, Jiangsu 210023, China; ^2^Brain Imaging Lab and MRI Unit, New York State Psychiatry Institute and Columbia University, New York, NY 10032, USA; ^3^School of Electronic Science and Engineering, Nanjing University, Nanjing, Jiangsu 210046, China

## Abstract

Automated abnormal brain detection is extremely of importance for clinical diagnosis. Over last decades numerous methods had been presented. In this paper, we proposed a novel hybrid system to classify a given MR brain image as either normal or abnormal. The proposed method first employed digital wavelet transform to extract features then used principal component analysis (PCA) to reduce the feature space. Afterwards, we constructed a kernel support vector machine (KSVM) with RBF kernel, using particle swarm optimization (PSO) to optimize the parameters *C* and **σ**. Fivefold cross-validation was utilized to avoid overfitting. In the experimental procedure, we created a 90 images dataset *brain* downloaded from Harvard Medical School website. The abnormal brain MR images consist of the following diseases: glioma, metastatic adenocarcinoma, metastatic bronchogenic carcinoma, meningioma, sarcoma, Alzheimer, Huntington, motor neuron disease, cerebral calcinosis, Pick's disease, Alzheimer plus visual agnosia, multiple sclerosis, AIDS dementia, Lyme encephalopathy, herpes encephalitis, Creutzfeld-Jakob disease, and cerebral toxoplasmosis. The 5-folded cross-validation classification results showed that our method achieved 97.78% classification accuracy, higher than 86.22% by BP-NN and 91.33% by RBF-NN. For the parameter selection, we compared PSO with those of random selection method. The results showed that the PSO is more effective to build optimal KSVM.

## 1. Introduction

Magnetic resonance imaging (MRI) is an imaging technique that produces high quality images of the anatomical structures of the human body, especially in the brain, and provides rich information for clinical diagnosis and biomedical research. The diagnostic values of MRI are greatly magnified by the automated and accurate classification of the MRI images.

Wavelet transform is an effective tool for feature extraction from MR brain images, because they allow analysis of images at various levels of resolution due to its multiresolution analytic property. However, this technique requires large storage and is computationally expensive [1]. In order to reduce the feature vector dimensions and increase the discriminative power, the principal component analysis (PCA) has been used. PCA is appealing since it effectively reduces the dimensionality of the data and therefore reduces the computational cost of analyzing new data [2]. Then, the problem of how to classify on the input data comes.

In recent years, researchers have proposed a lot of approaches for this goal, which fall into two categories. One category is supervised classification, including support vector machine (SVM) [3] and *k*-nearest neighbors (*k*-NN) [4]. The other category is unsupervised classification, including self-organization feature map (SOFM) [3] and fuzzy *c*-means [5]. While all these methods achieved good results, yet the supervised classifier performs better than unsupervised classifier in terms of classification accuracy (success classification rate) [6].

Among supervised classification methods, the SVMs are state-of-the-art classification methods based on machine learning theory [7]. Compared with other methods such as artificial neural network, decision tree, and Bayesian network, SVMs have significant advantages of high accuracy, elegant mathematical tractability, and direct geometric interpretation. Besides, it does not need a large number of training samples to avoid overfitting [8]. 

Original SVMs are linear classifiers. In this paper, we introduced in the kernel SVMs (KSVMs), which extends original linear SVMs to nonlinear SVM classifiers by applying the kernel function to replace the dot product form in the original SVMs [9]. The KSVMs is allowed to fit the maximum-margin hyperplane in a transformed feature space. The transformation may be nonlinear, and the transformed space may be high dimensional; thus though the classifier is a hyperplane in the high-dimensional feature space, it may be nonlinear in the original input space [10].

The structure of the rest of this paper was organized as follows.  Section 2 gave the detailed procedures of preprocessing, including the discrete wavelet transform (DWT) and principal component analysis (PCA).  Section 3 first introduced the motivation and principles of linear SVM and then extended it to soft margin, dual from.  Section 4 introduced the method of PSO-KSVM. It first gave the principles of KSVM and then used the particle swarm optimization algorithm to optimize the values of parameters *C* and *σ*; finally it used *K*-fold cross-validation to protect the classifier from overfitting. The pseudocodes and flowchart were listed. Experiments in Section 5 created a dataset *brain* of 90 brain MR images and showed the results of each step. We compared our proposed PSO-KSVM method with traditional BP-NN and RBF-NN methods. Final Section 6 was devoted to conclusions and discussions.

## 2. Preprocessing

### 2.1. Feature Extraction

The most conventional tool of signal analysis is Fourier transform (FT), which breaks down a time domain signal into constituent sinusoids of different frequencies, thus transforming the signal from time domain to frequency domain. However, FT has a serious drawback as discarding the time information of the signal. For example, analyst cannot tell when a particular event took place from a Fourier spectrum. Thus, the classification will decrease as the time information is lost.

Gabor adapted the FT to analyze only a small section of the signal at a time. The technique is called windowing or short-time Fourier transform (STFT) [11]. It adds a window of particular shape to the signal. STFT can be regarded as a compromise between the time information and frequency information. It provides some information about both time and frequency domain. However, the precision of the information is limited by the size of the window.

**Figure 1:**
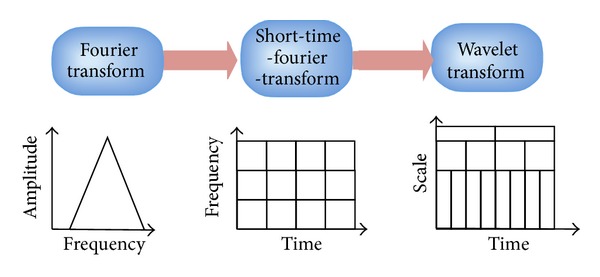
The development of signal analysis.

Wavelet transform (WT) represents the next logical step: a windowing technique with variable size. Thus, it preserves both time and frequency information of the signal. The development of signal analysis is shown in Figure 1.

Another advantage of WT is that it adopts “scale” instead of traditional “frequency,” namely, it does not produce a time-frequency view but a time-scale view of the signal. The time-scale view is a different way to view data, but it is a more natural and powerful way.

### 2.2. Discrete Wavelet Transform

The discrete wavelet transform (DWT) is a powerful implementation of the WT using the dyadic scales and positions. The basic fundamental of DWT is introduced as follows. Suppose that *x*(*t*) is a square-integrable function, then the continuous WT of *x*(*t*) relative to a given wavelet *ψ*(*t*) is defined as

(1) $$\begin{array}{c} W_{\psi }\left(a,b\right)=\int_{-\infty }^{\infty }x\left(t\right)\psi_{a,b}\left(t\right)dt, \end{array}$$

where

(2) $$\begin{array}{c} \psi_{a,b}\left(t\right)=\frac{1}{\sqrt{a}}\psi \left(\frac{t-a}{b}\right). \end{array}$$

Here, the wavelet *ψ*_*a*,*b*_(*t*) is calculated from the mother wavelet *ψ*(*t*) by translation and dilation: *a* is the dilation factor, and *b* is the translation parameter (both real positive numbers). There are several different kinds of wavelets which have gained popularity throughout the development of wavelet analysis. The most important wavelet is the Harr wavelet, which is the simplest one and often the preferred wavelet in a lot of applications.

Equation (1) can be discretized by restraining *a* and *b* to a discrete lattice (*a* = 2^*b*^ & *a* > 0) to give the DWT, which can be expressed as follows:

(3) caj,k(n)=DS[∑nx(n)gj∗(n−2jk)],cdj,k(n)=DS[∑nx(n)hj∗(n−2jk)].

Here ca_*j*,*k*_ and cd_*j*,*k*_ refer to the coefficients of the approximation components and the detail components, respectively. *g*(*n*) and *h*(*n*) denote the low-pass filter and high-pass filter, respectively. *j* and *k* represent the wavelet scale and translation factors, respectively. DS operator means the downsampling.

**Figure 2:**
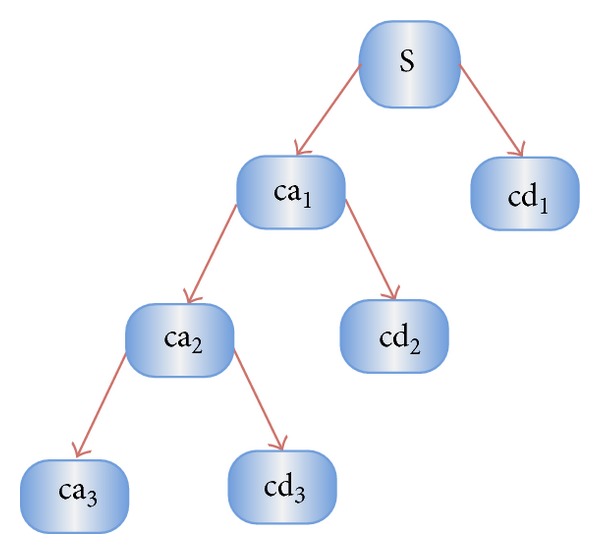
A 3-level wavelet decomposition tree.

The above decomposition process can be iterated with successive approximations being decomposed in turn, so that one signal is broken down into various levels of resolution. The whole process is called wavelet decomposition tree, shown in Figure 2.

### 2.3. 2D DWT

**Figure 3:**
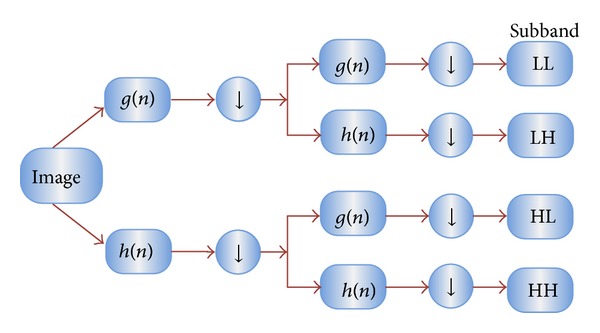
Schematic diagram of 2D DWT.

In case of 2D images, the DWT is applied to each dimension separately.  Figure 3 illustrates the schematic diagram of 2D DWT. As a result, there are 4 subband (LL, LH, HH, and HL) images at each scale. The sub-band LL is used for the next 2D DWT. 

The LL subband can be regarded as the approximation component of the image, while the LH, HL, and HH subbands can be regarded as the detailed components of the image. As the level of decomposition increased, compacter but coarser approximation component was obtained. Thus, wavelets provide a simple hierarchical framework for interpreting the image information. In our algorithm, level 3 decomposition via Harr wavelet was utilized to extract features. 

### 2.4. Feature Reduction

Excessive features increase computation times and storage memory. Furthermore, they sometimes make classification more complicated, which is called the curse of dimensionality. It is required to reduce the number of features [12].

PCA is an efficient tool to reduce the dimension of a data set consisting of a large number of interrelated variables while retaining most of the variations. It is achieved by transforming the data set to a new set of ordered variables according to their variances or importance. This technique has three effects: it orthogonalizes the components of the input vectors, so that it uncorrelated with each other, it orders the resulting orthogonal components, so that those with the largest variation come first, and it eliminates those components contributing the least to the variation in the data set.

It should be noted that the input vectors should be normalized to have zero mean and unity variance before performing PCA. The normalization is a standard procedure. Details about PCA could be seen in [13].

## 3. SVM Classifier

The introduction of support vector machine (SVM) is a landmark of the field of machine learning [14]. The advantages of SVMs include high accuracy, elegant mathematical tractability and direct geometric interpretation [15]. Recently, multiple improved SVMs have grown rapidly, among which the kernel SVMs are the most popular and effective. Kernel SVMs have the following advantages [16]: (1) work very well in practice and have been remarkably successful in such diverse fields as natural language categorization, bioinformatics, and computer vision; (2) have few tunable parameters; and (3) training often employs convex quadratic optimization [17]. Hence, solutions are global and usually unique, thus avoiding the convergence to local minima exhibited by other statistical learning systems, such as neural networks.

### 3.1. Principles of Linear SVMs

**Figure 4:**
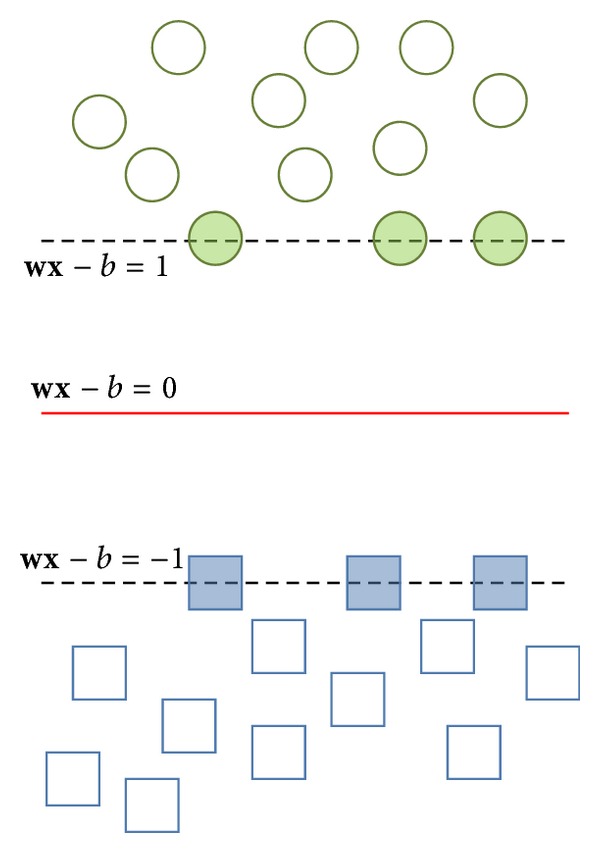
The concept of parallel hyperplanes.

Given a *p*-dimensional training dataset of size *N*in the form

(4) $$\begin{array}{c} \left\{\left(x_{n},y_{n}\right)\;|\;x_{n}\in R^{p},y_{n}\in \left\{-1,+1\right\}\right\},\;n=1,\ldots ,N, \end{array}$$

where *y*_*n*_ is either −1 or 1 corresponding to the class 1 or 2. Each *x*_*n*_ is a *p*-dimensional vector. The maximum-margin hyperplane which divides class 1 from class 2 is the support vector machine we want. Considering that any hyperplane can be written in the form of

(5) $$\begin{array}{c} wx-b=0, \end{array}$$

where · denotes the dot product and **w** denotes the normal vector to the hyperplane. We want to choose the **w** and *b* to maximize the margin between the two parallel (as shown in Figure 4) hyperplanes as large as possible while still separating the data. So we define the two parallel hyperplanes by the equations as

(6) $$\begin{array}{c} wx-b=\pm 1. \end{array}$$

Therefore, the task can be transformed to an optimization problem. That is, we want to maximize the distance between the two parallel hyperplanes, subject to prevent data falling into the margin. Using simple mathematical knowledge, the problem can be finalized as

(7) min⁡w,b||w||s.t.  yn(wxn−b)≥1, n=1,…,N.

In practical situations the ||**w**|| is usually replaced by

(8) min⁡w,b12||w||2s.t.  yn(wxn−b)≥1, n=1,…,N.

The reason leans upon the fact that ||**w**|| is involved in a square root calculation. After it is superseded with formula (8), the solution will not change, but the problem is altered into a quadratic programming optimization that is easy to solve by using Lagrange multipliers and standard quadratic programming techniques and programs.

### 3.2. Soft Margin

However, in practical applications, there may exist no hyperplane that can split the samples perfectly. In such case, the “*soft margin*” method will choose a hyperplane that splits the given samples as clean as possible, while still maximizing the distance to the nearest cleanly split samples. 

Positive slack variables *ξ*_*n*_ are introduced to measure the misclassification degree of sample *x*_*n*_ (the distance between the margin and the vectors *x*_*n*_ that lying on the wrong side of the margin). Then, the optimal hyperplane separating the data can be obtained by the following optimization problem:

(9) min⁡w,ξ,b  12||w||2+C∑n=1Nξns.t.{yn(wxn−b)≥1−ξnξn≥0,n=1,…,N,

where *C* is the error penalty. Therefore, the optimization becomes a tradeoff between a large margin and a small error penalty. The constraint optimization problem can be solved using “*Lagrange multiplier*” as

(10) min⁡w,ξ,b max⁡α,β{12||w||2+C∑n=1Nξn−∑n=1Nαn[yn(wxn−b)−1+ξn]−∑n=1Nβnξn}.

The min-max problem is not easy to solve, so Cortes and Vapnik proposed a *dual form* technique to solve it. 

### 3.3. Dual Form

The dual form of formula (9) can be designed as

(11) max⁡α∑n=1Nαn−12∑n=1N ∑m=1Nαmαnymynk(xm,xn),s.t.{0≤αn≤C,∑n=1Nαnyn=0,n=1,…,N.

The key advantage of the dual form function is that the slack variables *ξ*_*n*_ vanish from the dual problem, with the constant *C* appearing only as an additional constraint on the Lagrange multipliers. Now, the optimization problem (11) becomes a *quadratic programming* (QP) problem, which is defined as the optimization of a quadratic function of several variables subject to linear constraints on these variables. Therefore, numerous methods can solve formula (9) within milliseconds, like interior point method, active set method, augmented Lagrangian method, conjugate gradient method, simplex algorithm, and so forth.

## 4. PSO-KSVM

### 4.1. Kernel SVMs

Linear SVMs have the downside to linear hyperplane, which cannot separate complicated distributed practical data. In order to generalize it to nonlinear hyperplane, the kernel trick is applied to SVMs [18]. The resulting algorithm is formally similar, except that every dot product is replaced by a nonlinear kernel function. In another point of view, the KSVMs allow to fit the maximum-margin hyperplane in a transformed feature space. The transformation may be nonlinear, and the transformed space may be higher dimensional; thus though the classifier is a hyperplane in the higher-dimensional feature space, it may be nonlinear in the original input space. For each kernel, there should be at least one adjusting parameter so as to make the kernel flexible and tailor itself to practical data. In this paper, RBF kernel is chosen due to its excellent performance. The kernel is written as

(12) $$\begin{array}{c} k\left(x_{m},x_{n}\right)=\exp\left(-\frac{||x_{m}-x_{n}||}{2\sigma^{2}}\right). \end{array}$$

Put formula (12) into formula (11), and we got the final SVM training function as

(13) max⁡α∑n=1Nαn−12∑n=1N ∑m=1Nαmαnymynexp⁡(−||xm−xn||2σ2),s.t.{0≤αn≤C,∑n=1Nαnyn=0,n=1,…,N.

It is still a quadratic programming problem, and we chose interior point method to solve the problem. However, there is still an outstanding issue, that is, the value of parameters *C* and *σ* in (13).

### 4.2. PSO

To determine the best parameter of *C* and *σ*, traditional method uses trial-and-error methods. It will cause heavy computation burden and cannot guarantee to find the optimal or even near-optimal solutions. Fei, W. [19] and Chenglin et al. [20] proposed to use PSO to optimize the parameters, respectively, and independently. The PSO is a populated global optimization method, deriving from the research of the movement of bird flocking or fish schooling. It is easy and fast to implement. Besides, we introduced in the cross-validation to construct the fitness function used for PSO.

PSO performs search via a swarm of particles which is updated from iteration to iteration. To seek for the optimal solution, each particle moves in the direction of its previously best position (*p*_best_) and the best global position in the swarm (*g*_best_) as follows:

(14) pbesti=pi(k∗)s.t.  fitness(pi(k∗))=min⁡k=1,…,t[fitness(pi(k))],gbest=pi∗(k∗)s.t.  fitness(pi∗(k∗))=min⁡i=1,…,Pk=1,…,t[fitness(pi(k))],

where *i* denotes the particle index, *P* denotes the total number of particles, *k* denotes the iteration index, and *t* denotes the current iteration number, and *p* denotes the position. The velocity and position of particles can be updated by the following equations:

(15) vi(t+1)=wvi(t)+c1r1(pbesti(t)−pi(t))+c2r2(gbest(t)−pi(t)),pi(t+1)=pi(t)+vi(t+1),

where *v* denotes the velocity. The inertia weight *w* is used to balance the global exploration and local exploitation. The *r*_1_ and *r*_2_ are uniformly distributed random variables within range (0, 1). The *c*_1_ and *c*_2_ are positive constant parameters called “acceleration coefficients.” Here, the particle encoding is composed of the parameters *C* and *σ* in (13).

### 4.3. Cross-Validation

In this paper we choose 5-fold considering the best compromise between computational cost and reliable estimates. The dataset is randomly divided into 5 mutually exclusively subsets of approximately equal size, in which 4 subsets are used as training set, and the last subset is used as validation set. The abovementioned procedure repeated 5 times, so each subset is used once for validation. The fitness function of PSO chose the classification accuracy of the 5-fold cross-validation:

(16) $$\begin{array}{c} \text{fitness}=\frac{1}{5}\sum_{i=1}^{5}\left|\frac{y_{s}}{y_{s}+y_{m}}\right|. \end{array}$$

Here *y*_*s*_ and *y*_*m*_ denote the number of successful classification and misclassification, respectively. PSO is performed to maximize the fitness function (classification accuracy).

### 4.4. Pseudocodes of Our Method

**Figure 5:**
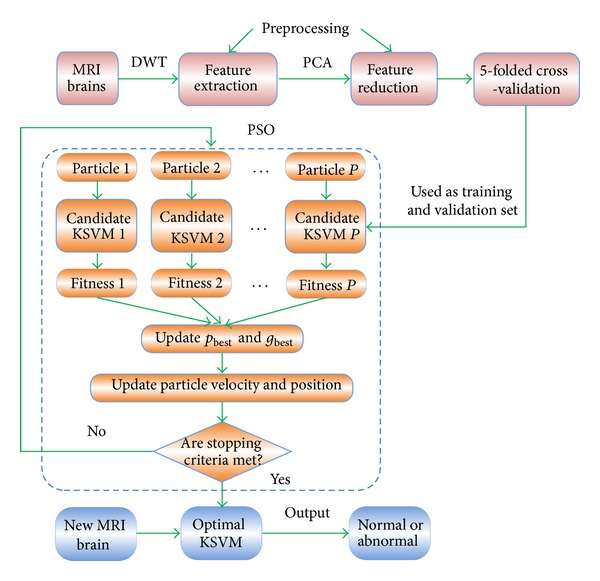
Methodology of our proposed PSO-KSVM algorithm.

In total, our method can be described as the following three stages, and the flowchart is depicted in Figure 5.

Step 1: Collecting MR brain images dataset.
Step 2: Preprocessing (including feature extraction and feature reduction).
Step 3: Fivefolded cross-validation.
Step 4: Determining the best parameter.
  Step 4.1: Initializing PSO. The particles correspond to *C* and *σ*.
  Step 4.2: For each particle *i*, computer the fitness values.
    Step 4.2.1: Decoding the particle to parameters *C* and *σ*.
    Step 4.2.2: Using interior method to train KSVM according to (13).
    Step 4.2.3: Calculating classification error according to (16) as the fitness values.
  Step 4.3: Updating the *g*_best_ and *p*_best_ according to (14).
  Step 4.4: Updating the velocity and position of each particle according to (15).
  Step 4.5: If stopping criteria is met, then jump to Step 4.6; otherwise return to Step 4.2.
  Step 4.6: Decoding the optimal particle to corresponding parameter C* and σ*.
Step 5: Constructing KSVM via the optimal C* and σ* according to (13).
Step 6: Submitting new MRI brains to the trained KSVM and outputting the prediction.

## 5. Experiments and Discussions

The experiments were carried out on the platform of P4 IBM with 3.3 GHz processor and 2 GB RAM, running under Windows XP operating system. The algorithm was in-house developed via the wavelet toolbox, the biostatistical toolbox of 32 bit MATLAB 2012a (the MathWorks). The programs can be run or tested on any computer platforms where MATLAB is available.

### 5.1. Database

**Figure 6:**
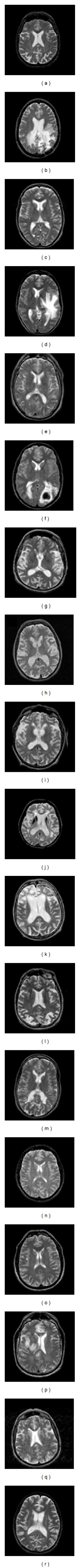
Sample of brain MRIs: (a) normal brain; (b) glioma, (c) metastatic adenocarcinoma; (d) metastatic bronchogenic carcinoma; (e) meningioma; (f) sarcoma; (g) Alzheimer; (h) Huntington; (i) motor neuron disease; (j) cerebral calcinosis; (k) Pick's disease; (l) Alzheimer plus visual agnosia; (m) multiple sclerosis; (n) AIDS dementia; (o) Lyme encephalopathy; (p) herpes encephalitis; (q) Creutzfeld-Jakob disease; and (r) cerebral toxoplasmosis.

The datasets *brain* consists of 90 T2-weighted MR brain images in axial plane and 256 × 256 in-plane resolution, which were downloaded from the website of Harvard Medical School (URL: http://www.med.harvard.edu/aanlib/home.html). The abnormal brain MR images of the dataset consist of the following diseases: glioma, metastatic adenocarcinoma, metastatic bronchogenic carcinoma, meningioma, sarcoma, Alzheimer, Huntington, motor neuron disease, cerebral calcinosis, Pick's disease, Alzheimer plus visual agnosia, multiple sclerosis, AIDS dementia, Lyme encephalopathy, herpes encephalitis, Creutzfeld-Jakob disease, and cerebral toxoplasmosis. The samples of each disease are illustrated in Figure 6.

We randomly selected 5 images for each type of brain. Since there are 1 type of normal brain and 17 types of abnormal brain in the dataset, 5*(1 + 17) = 90 images were selected to construct the *brain* dataset, consisting of 5 normal and 85 abnormal brain images in total. 

**Figure 7:**
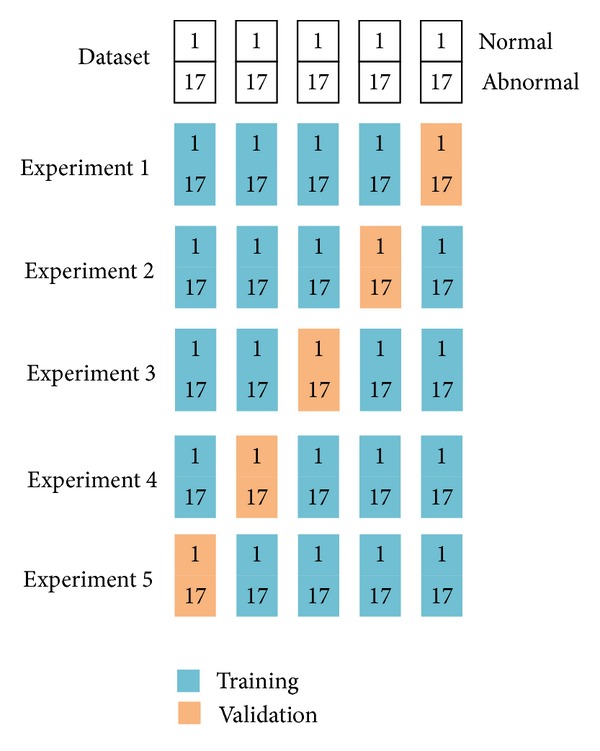
Illustration of 5-fold cross-validation of *brain* dataset (we divided the dataset into 5 groups, and for each experiment, 4 groups were used for training, and the rest one group was used for validation. Each group was used once for validation).

The setting of the training images and validation images was shown in Figure 7. We divided the dataset into 5 equally distributed groups; each groups contain one normal brain and 17 abnormal brains. Since 5-fold cross-validation was used, we would perform 5 experiments. In each experiment, 4 groups were used for training, and the left 1 group was used for validation. Each group was used once for validation. In total, in this cross validation way, 360 images were for training, and 90 images were for validation.

### 5.2. Feature Extraction

**Figure 8:**
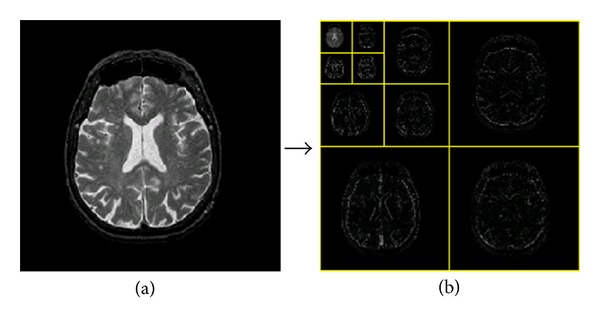
The procedures of 3-level 2D DWT: (a) normal brain MRI; (b) level 3 wavelet coefficients.

The three levels of wavelet decomposition greatly reduce the input image size as shown in Figure 8. The top left corner of the wavelet coefficients image denotes for the approximation coefficients at level 3, of which the size is only 32 × 32 = 1024. The border distortion should be avoided. In our algorithm, symmetric padding method [21] was utilized to calculate the boundary value.

### 5.3. Feature Reduction

**Figure 9:**
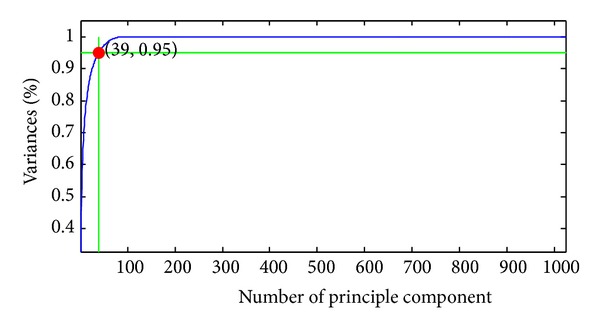
The curve of variances against number of principle components (here we found that 39 features can achieve 95.02% variances).

As stated above, the extracted features were reduced from 65536 to 1024 by the DWT procedure. However, 1024 was still too large for calculation. Thus, PCA was used to further reduce the dimensions of features. The curve of cumulative sum of variance versus the number of principle components was shown in Figure 9. 

The variances versus the number of principle components from 1 to 40 were listed in Table 1. It showed that only 39 principle components (bold font in table), which were only 39/1024 = 3.81% of the original features, could preserve 95.02% of total variance.

### 5.4. Classification Accuracy

The KSVM used the RBF as the kernel function. We compared our PSO-KVSM method with one hidden-layer Back Propagation-Neural Network (BP-NN) and RBF-Neural Network (RBF-NN). The results were shown in Table 2. It showed that BP-NN correctly matched 388 cases with 86.22% classification accuracy. RBF-NN correctly matched 411 cases with 91.33% classification accuracy. Our PSO-KSVM correctly matched 440 brain images with 97.78% classification accuracy. Therefore, our method had the most excellent classification performance.

### 5.5. Parameter Selection

The final parameters obtained by PSO were *C* = 143.3 and *σ* = 1.132. We compared this case with random selection method, which randomly generated the values of *C* in the range of (50, 200) and *σ* in the range of [0.5, 2], and then we compared them with the optimized values by PSO (*C* = 143.3 and *σ* = 1.132). The results achieved by random selection method were shown in Table 3. We saw that the classification accuracy varied with the change of parameters *σ* and *C*, so it was important to determine the optimal values before constructing the classifier. The random selection method was difficult to come across the best values, so PSO was an effective method for this problem compared to random selection method.

## 6. Conclusions and Discussions

In this study we had developed a novel DWT + PCA + PSO-KSVM hybrid classification system to distinguish between normal and abnormal MRIs of the brain. We picked up RBF as the kernel function of SVM. The experiments demonstrated that the PSO-KSVM method obtained 97.78% classification accuracy on the 5-folded 90-image dataset, higher than 86.22% of BP-NN and 91.33% of RBF-NN.

Future work should focus on the following four aspects. First, the proposed SVM based method could be employed for MR images with other contrast mechanisms such as T1-weighted, proton density weighted, and diffusion weighted images. Second, the computation time could be accelerated by using advanced wavelet transforms such as the lift-up wavelet. Third, Multiclassification, which focuses on brain MRIs of specific disorders, can also be explored. Forth, novel kernels will be tested to increase the classification accuracy and accelerate the algorithm.

The DWT can efficiently extract the information from original MR images with litter loss. The advantage of DWT over Fourier transforms is the spatial resolution; namely, DWT captures both frequency and location information. In this study we choose the Harr wavelet, although there are other outstanding wavelets such as Daubechies series. We will compare the performance of different families of wavelet in future work. Another research direction lies in the stationary wavelet transform and the wavelet packet transform.

The importance of PCA was demonstrated in the Discussion. If we omitted the PCA procedures, we meet a huge feature space (1024 dimensions) which will cause heavy computation burden and lowered the classification accuracy. There are some other excellent feature reduction methods such as ICA, manifold learning. In the future, we will focus on investigating the performance of those algorithms. 

The reason we choose RBF kernel is that RBF takes the form of exponential function, which enlarge the sample distances to the uttermost extent. In the future, we will try to test other kernel functions.

The importance of introducing PSO is to determine the optimal values of parameters *C* and *σ*. From random selection method, we found it is hard to get the optimal values at the parameter space. Therefore, the PSO is an effective way to find the optimal values. Integrating PSO to KSVM enhance the classification capability of KSVM.

The most important contribution of this paper is the propose of a hybrid system, integrating DWT, PCA, PSO, KSVM, and CV, used for identifying normal MR brains from abnormal MR brains. It would be useful to help clinicians to diagnose the patients.

## Figures and Tables

**Table 1:** Detailed data of PCA.

Number of prin. comp.	1	2	3	4	5	6	7	8	9	10
variance (%)	32.81	44.53	52.35	57.71	61.97	65.02	67.78	70.18	72.46	74.56

Number of prin. comp.	11	12	13	14	15	16	17	18	19	20
variance (%)	76.41	78.2	79.7	81.12	82.27	83.38	84.35	85.29	86.05	86.8

Number of prin. comp.	21	22	23	24	25	26	27	28	29	30
variance (%)	87.53	88.2	88.8	89.35	89.86	90.35	90.84	91.3	91.73	92.15

Number of prin. comp.	31	32	33	34	35	36	37	38	**39**	40
variance (%)	92.54	92.9	93.24	93.58	93.9	94.21	94.5	94.76	**95.02**	95.27

**Table 2:** Methods of comparison between BP-NN, RBF-NN, and PSO-KSVM.

Method	Confusion matrix	Success Cases	Sensitivity	Specificity	Classification accuracy
BP-NN	$\begin{array}{cc} 374 & 11 \end{array}\;$ $\begin{array}{cc} 51 & 14 \end{array}$	388	88.0%	56%	86.22%
RBF-NN	$\begin{array}{cc} 393 & 7 \end{array}\;$ $\begin{array}{cc} 32 & 18 \end{array}$	411	92.47%	72%	91.33%
PSO-KSVM	$\begin{array}{cc} 417 & 2 \end{array}\;\;$ $\;\begin{array}{cc} 8 & 23 \end{array}$	440	98.12%	92%	97.78%

**Table 3:** Parameters of comparison by random selection method (the final row corresponds to our proposed method).

	*σ*	*C*	Success case	Classification accuracy
Random 1	0.625	124.71	410	91.11%
Random 2	1.439	185.13	412	91.56%
Random 3	1.491	136.20	423	94.00%
Random 4	1.595	176.78	409	90.89%
Random 5	1.836	160.80	401	89.11%
Random 6	1.973	137.90	401	89.11%
Random 7	1.654	87.01	396	88.00%
Random 8	1.372	149.96	427	94.89%

Optimized	1.132	143.3	440	97.78%
